# Thermal Decomposition Characteristics of Silicone Rubber in In-Service Power Equipment: A Study Combining Experiments and Molecular Simulations

**DOI:** 10.3390/polym18091137

**Published:** 2026-05-06

**Authors:** Jiaming Yan, Runmiao Shi, Zhijun An, Haoran Meng, Xinhan Qiao, Wenyu Ye

**Affiliations:** 1 School of Electrical Engineering, China University of Mining and Technology, Xuzhou 221116, China; yanjiaming888@163.com (J.Y.); ts25230059a31@cumt.edu.cn (R.S.); ts24230001a31@cumt.edu.cn (Z.A.); ts24230031a31@cumt.edu.cn (H.M.); qiaoxinhan@cumt.edu.cn (X.Q.); 2 State Key Laboratory of Power Transmission Equipment Technology, School of Electrical Engineering, Chongqing University, Chongqing 400044, China

**Keywords:** silicone rubber, thermal decomposition, reactive molecular dynamics simulation, thermodynamic properties

## Abstract

To investigate the thermal decomposition characteristics of high-temperature vulcanized silicone rubber (HTV) and liquid silicone rubber (LSR) under different aging conditions, scanning electron microscopy (SEM) and thermogravimetric analysis (TG) were employed to characterize the surface microstructure and chemical properties of silicone rubber samples that had been in service for 15 years. The influence of aging degree on the thermal stability of silicone rubber was initially investigated. ReaxFF-based reactive molecular dynamics simulations were conducted to analyze the decomposition pathways of silicone rubber under high-temperature conditions, as well as the dynamic evolution of decomposition products. In addition, key parameters—including glass transition temperature, mean square displacement, cohesive energy density, and free volume fraction—were calculated before and after decomposition using the Materials Studio platform. The results indicate that LSR exhibits higher thermal stability than HTV, while the thermal stability of both materials decreases after thermal decomposition. Furthermore, the variation in thermal stability was discussed based on these parameters from the perspectives of molecular mobility and intermolecular interactions. This research can provide a reference for the safety operation assessment, aging status determination, and high-temperature service reliability design of silicone rubber insulating materials.

## 1. Introduction

Silicone rubber is extensively utilized in high-end applications, including power equipment, aerospace, and electronic devices, owing to its excellent high-temperature resistance, aging resistance, superior insulation performance, and stable mechanical properties [1,2]. However, under the combined influence of complex environmental stressors—such as moisture ingress, atmospheric weathering, ultraviolet radiation, and elevated temperatures—silicone rubber used for external insulation is susceptible to surface cracking, hardening, and embrittlement [3]. These degradation processes lead to a decline in material performance, reduced service life, and potential safety risks [4,5]. Therefore, a comprehensive investigation into the aging and thermal decomposition mechanisms of silicone rubber under high-temperature conditions, as well as its thermal stability behavior, is of significant theoretical and engineering importance for extending service life and ensuring the safe and reliable operation of equipment.

Thermal stability is one of the most crucial performance indicators for silicone rubber in outdoor insulation, high-voltage electrical equipment, and long-term service environments. Silicone rubber can be broadly classified into high-temperature vulcanized silicone rubber (HTV) and liquid silicone rubber (LSR) [6]. As the two most widely used types, their thermal stability may differ significantly due to variations in preparation processes and molecular structures [7,8]. Existing studies, both domestic and international, have predominantly focused on either macroscopic characterization or purely molecular-level simulations when investigating the thermal stability of silicone rubber. For instance, Chen C et al. [9] conducted macroscopic experiments and qualitatively explained from the perspectives of crosslinking and fillers, characterizing and analyzing the thermal oxidative aging behavior of outdoor insulation HTV silicone rubber. This belongs to experimental testing research. While Chenoweth K et al. [10] used ReaxFF reactive stress field to conduct molecular simulation on the thermal decomposition of PDMS, which belongs to research at the molecular simulation level. At the macroscopic level, tests such as thermogravimetric analysis are often used to explore the weight loss patterns, thermal decomposition rates and other characteristics of materials [11], and to directly evaluate the differences in thermal stability. However, it is difficult to reveal the microscopic cracking reaction pathways and product evolution mechanisms [12]. At the microscopic level, although molecular simulation methods can be used to analyze the essence of the reaction [13], the simulation results lack validation by macroscopic experimental data. These research limitations have led to the fact that the intrinsic reasons for the differences in thermal stability of the two types of silicone rubbers have not been systematically clarified.

Thermogravimetric analysis (TGA), as a classical macroscopic technique for evaluating material thermal stability, enables quantitative assessment through key parameters such as the 5% weight loss temperature, the temperature at the maximum decomposition rate, and the final residue fraction [14]. In contrast, reaction field–based molecular dynamics simulation using the ReaxFF force field (ReaxFF MD) provides atomic-scale insight into the chemical reactions and structural evolution of polymers under thermal, mechanical, and environmental stimuli. This approach facilitates the elucidation of bond scission pathways and product evolution mechanisms, thereby offering mechanistic support for macroscopic experimental observations [15]. Furthermore, the integration of thermodynamic parameters—including the glass transition temperature (Tg), mean square displacement (MSD), cohesive energy density (CED), and free volume fraction (FFV) [16]—enables a more comprehensive understanding of the intrinsic factors governing changes in thermal stability.

This study investigates the thermal stability differences between HTV and LSR silicone rubber through macroscopic characterization tests and ReaxFF molecular dynamics simulations. The thermal stability of samples with varying aging degrees was examined, revealing differences between the two types of silicone rubber. The cracking reaction pathways and the dynamic evolution of product quantities were explored using ReaxFF MD simulations. By integrating thermodynamic indicators, the underlying mechanisms governing changes in thermal stability were analyzed, indicating the fundamental causes of the thermal stability differences between HTV and LSR, as well as the impact of aging on their thermal stability. These findings provide theoretical insights and experimental data for optimizing performance and engineering applications of silicone rubber materials.

## 2. Test Results and Analysis of On-Site Running Bushing Silicone Rubber Samples

### 2.1. Experimental Sample

**Figure 1:**
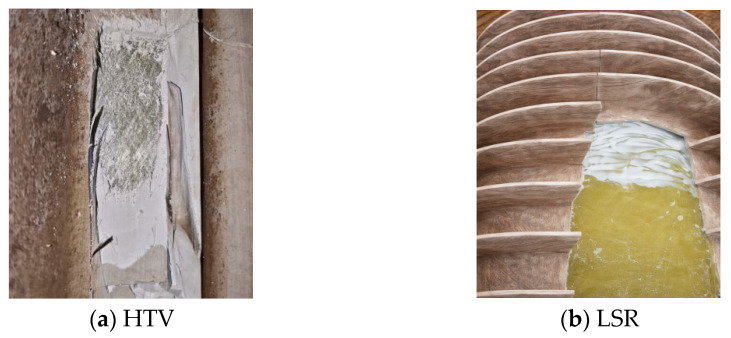
Schematic diagram of on-site sampling of silicone rubber samples.

The HTV silicone rubber test samples were taken from a 1000 kV high-temperature cured silicone rubber bushing that had been in service for 15 years at the site. This bushing was from the northern part of the Yangtze River Delta in the subtropical monsoon climate zone and was a precious carrier for studying the performance evolution laws of materials under real working conditions. The on-site sampling photos of the HTV silicone rubber samples are shown in Figure 1a.

The LSR silicone rubber test samples were taken from a 550 kV liquid silicone rubber bushing that had been in service for 15 years at the site. This bushing was installed vertically in the system, with the high-pressure end at the top and the low-pressure end at the bottom. The on-site sampling photos of the LSR silicone rubber samples are shown in Figure 1b.

Due to the non-uniform electric field distribution along different positions of the casing body and umbrella skirts, the material exhibits spatially non-uniform aging characteristics during operation, resulting in significant variations in aging degree at different locations. Accordingly, HTV and LSR silicone rubber samples with different aging levels were selected from various positions to systematically investigate their thermal decomposition behavior under different aging conditions. The aging degree of the silicone rubber samples was evaluated based on multiple quantitative characterization methods, including electric field intensity distribution, SEM surface morphology, FTIR spectra, EDS elemental composition, and TG thermal analysis. Detailed information of each sampling position is provided in Table 1. The aging degrees mild and severe were determined based on the combined variation in thermogravimetric parameters, including the 5 percent weight loss temperature and the temperature at the maximum decomposition rate, with surface morphology serving as supporting evidence. Samples exhibiting only slight reductions in thermal characteristic temperatures within experimental deviation and minor surface defects were classified as mildly aged. In contrast, samples showing significant decreases in both the initial decomposition temperature and the maximum decomposition rate temperature exceeding typical variation ranges, together with evident surface cracking and deterioration, were classified as severely aged.

### 2.2. Analysis of Characterization Test Results for Silicone Rubber Samples

**Figure 2:**
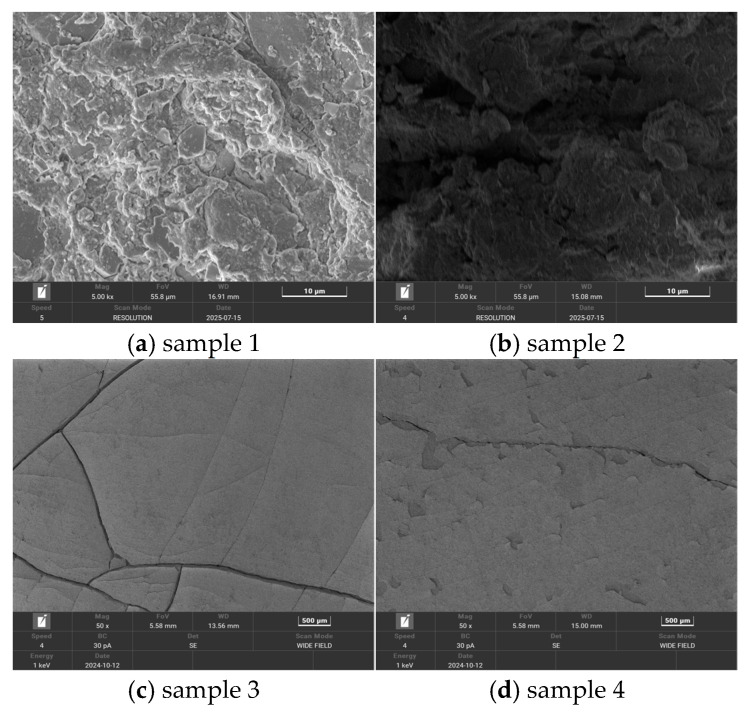
The microscopic surface morphology of the silicone rubber sample.

**Figure 3:**
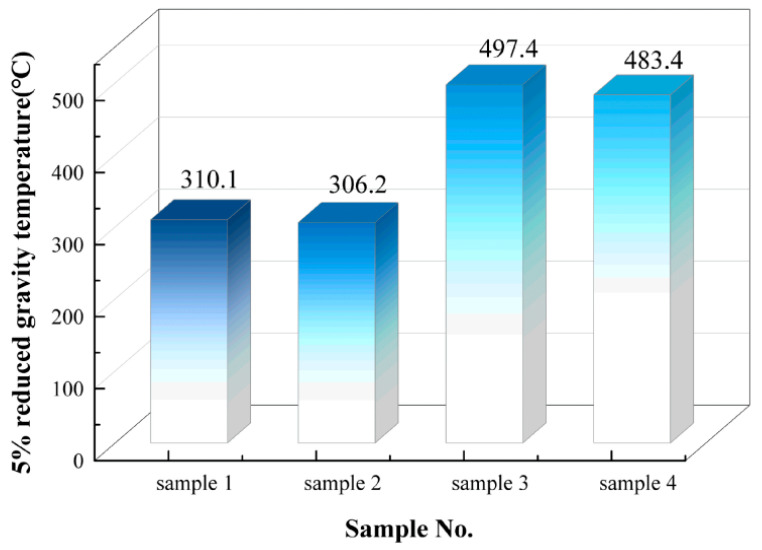
Statistical chart of 5% weight loss temperatures for four samples.

To deeply investigate the thermal stability and thermal decomposition behavior of HTV silicone rubber and LSR silicone rubber during aging, this paper conducted scanning electron microscopy and thermogravimetric analysis tests on the aged samples of the two types of silicone rubber. The surface microstructure of the samples was characterized using the TESCAN MIRA scanning electron microscope, and the testing process was carried out in accordance with the common testing specifications for characterizing the surface morphology of materials by the scanning electron microscope. The imaging signal was obtained through the interaction between the electron beam and the sample surface, allowing for the acquisition of surface morphology and roughness features at the micrometer and even nanometer scale, which were used to analyze the microscopic surface features of the samples. The thermogravimetric test used the Thermo plus EVO2 TG-DTA8121 type thermogravimetric analyzer. The testing process was protected by nitrogen filling of the sample space, with the test temperature ranging from 25 to 800 °C and the heating rate set at 30 °C/min. The thermogravimetric test analysis can conduct a systematic analysis of the differences in thermal decomposition characteristics of different types of silicone rubber. The entire testing process was carried out under an inert nitrogen atmosphere, effectively eliminating oxidation interference from oxygen and accurately reflecting the intrinsic thermal decomposition behavior of the silicone rubber matrix. This aligns with the molecular simulation conditions under inert environments described later, ensuring consistency between the macroscopic tests and the microscopic mechanism analysis.

(1). Figure 2 presents the microscopic surface morphologies of four silicone rubber samples. In this study, scanning electron microscopy was employed to qualitatively observe the surface morphology of the samples, providing a direct view of the surface deterioration characteristics under varying aging conditions. Quantitative analysis of parameters such as roughness, crack density, and defect ratio was not conducted. The difference in electron beam sensitivity between LSR and HTV is primarily governed by their microscopic cross-linking networks and aggregation structures. LSR, with its high degree of cross-linking and dense structure, exhibits significantly restricted chain segment mobility. As a result, molecular bond breakage and free radical defects induced by high-energy electron beams tend to accumulate continuously. In contrast, the more loosely structured network and larger chain segment mobility in HTV help buffer irradiation damage, reducing the extent of local structural degradation. Consequently, LSR demonstrates higher sensitivity to focused high-energy electron beams. For the HTV silicone rubber samples, which exhibit good resistance to electron beam irradiation, high-magnification imaging (5000×) was performed using an acceleration voltage of 5.00 kV, medium-speed scanning (Speed = 5), and high-resolution mode. This enabled clear observation of surface cracks and particle aggregation as the samples aged. SEM images of samples 1 and 2 revealed increased surface roughness, cracks, holes, and the accumulation of particle-like substances. A rougher surface, along with more prominent defects and cracks, indicates a more severe aging degree. For the more electron beam-sensitive LSR samples (samples 3 and 4), rapid carbonization and damage to the surface occurred under high-magnification imaging, preventing further detailed observation. To mitigate this issue, a lower acceleration voltage (1 kV), reduced beam current (30 pA), and faster scanning speed (Speed = 4) were employed, minimizing exposure time and reducing the risk of sample damage. Consequently, low-magnification (approximately 500×) imaging was consistently used to balance image clarity and minimize electron beam exposure. As shown in the figure, sample 3 exhibits numerous cracks, with some matrix particles showing signs of peeling, while sample 4 shows clear particle detachment, accompanied by deeper cracks and larger defects. The presented SEM images are representative of the overall sample morphology, and similar microstructural features were consistently observed across multiple regions of each sample.

(2). Figure 3 shows the statistical chart of the 5% weight loss temperatures for four silicone rubber samples. The 5% weight loss temperature represents the temperature at which the sample undergoes a 5% mass loss relative to its initial mass during the controlled heating process in thermogravimetric analysis. This parameter serves as a useful reference for evaluating a material’s resistance to thermal decomposition or volatile components. However, its interpretation must take into account the material’s formulation, composition, and the specific thermal stability characteristics of the silicone rubber. Under identical testing conditions, a higher 5% weight loss temperature indicates the material’s greater ability to maintain structural integrity at elevated temperatures and to resist thermal degradation. Thus, it can serve as a preliminary indicator of the material’s initial thermal stability.

**Figure 4:**
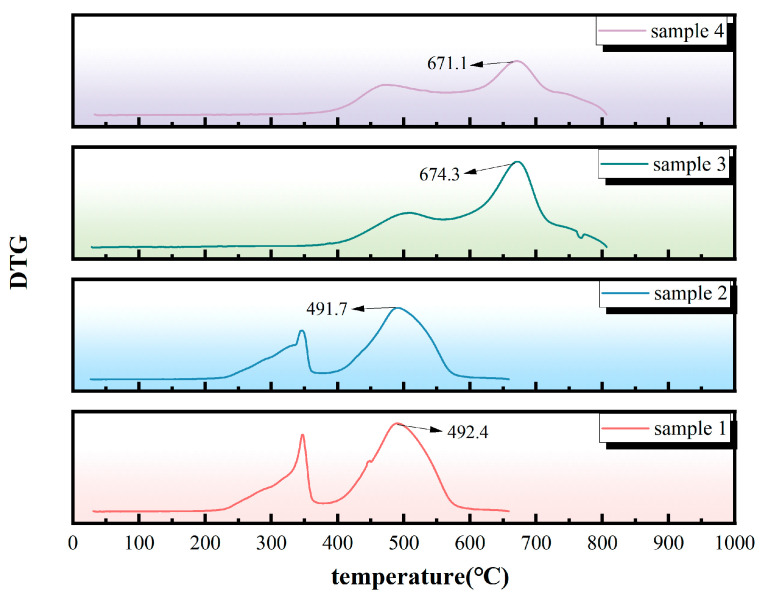
Graph of the maximum thermal decomposition rates for the four samples.

For samples 1 and 2, the 5% weight loss temperatures are relatively low, while samples 3 and 4 exhibit significantly higher temperatures. When considering differences in their formulations, it can be inferred that LSR silicone rubber demonstrates superior resistance to initial thermal decomposition and thus has better thermal stability than HTV silicone rubber. With increasing aging, sample 2 shows a decrease in the 5% weight loss temperature compared to sample 1, indicating a decline in HTV’s thermal stability with aging. Similarly, sample 4 exhibits a lower 5% weight loss temperature than sample 3, suggesting that LSR’s thermal stability also deteriorates over prolonged service. While these trends are generally consistent with expectations, a more comprehensive evaluation of thermal stability should consider the influence of factors such as the material’s composition, degree of crosslinking, and filler content, which can significantly affect the thermal decomposition behavior.

(3). Figure 4 shows the statistical graph of the maximum thermal decomposition rate temperatures for the four silicone rubber samples. The maximum thermal decomposition rate temperature corresponds to the peak temperature on the differential thermogravimetric curve, which characterizes the temperature at which the material exhibits the fastest rate of thermal decomposition. This parameter is an important reference for evaluating the thermal stability of materials. In general, a higher maximum thermal decomposition rate temperature indicates that the material can withstand higher temperatures before undergoing significant thermal decomposition, reflecting its overall resistance to thermal degradation. Given the inherent differences in the cross-linking structure, filler components, and matrix formulation between HTV and LSR silicone rubbers, these material properties directly influence the thermal decomposition kinetics and degradation behavior. Therefore, thermal stability should not be determined based on a single temperature indicator alone.

**Figure 5:**
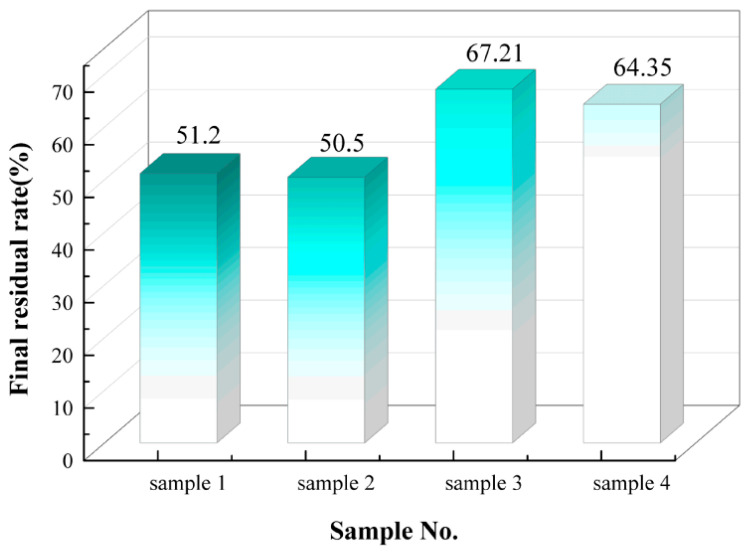
Statistical chart of the final residual rates of the four samples.

The maximum thermal decomposition rate temperatures of samples 1 and 2 are approximately 490 °C, whereas those of samples 3 and 4 are around 670 °C, significantly higher than those of samples 1 and 2. A comprehensive analysis of component differences suggests that LSR generally exhibits a superior ability to resist intense thermal decomposition. As aging progresses, the maximum thermal decomposition rate temperature of sample 2 decreases relative to sample 1, indicating that prolonged aging diminishes the thermal degradation resistance of HTV. Similarly, the maximum thermal decomposition rate temperature of sample 4 decreases compared to sample 3, suggesting that the thermal degradation resistance of LSR also deteriorates over extended field service.

(4). Figure 5 shows the statistical chart of the final residual rates of the four silicone rubber samples. The final residual rate refers to the percentage of the remaining mass relative to the initial mass when the sample’s mass no longer shows significant changes at the set termination temperature during thermogravimetric analysis. This parameter serves as an important reference for evaluating the high-temperature thermal degradation behavior of materials. The final residual rate is influenced not only by thermal stability but also by the matrix formulation, cross-linking structure, inorganic filler content, and the material’s carbonization behavior at high temperatures. Generally, under similar system conditions, a higher residual rate indicates that the material has experienced less thermal decomposition at high temperatures.

The final residual rates of samples 1 and 2 are approximately 50.5%, while those of samples 3 and 4 are around 65%, significantly higher than those of samples 1 and 2. A comprehensive analysis of the differences in the formulations and filler systems of the two types of silicone rubbers suggests that LSR exhibits lower high-temperature thermal decomposition loss and superior heat resistance compared to HTV. Comparing samples 1 and 2, it is evident that the final residual rate of sample 2 is lower. Similarly, comparing samples 3 and 4, the final residual rate of sample 4 is lower. These observations suggest that, as aging progresses, the silicone rubber matrix gradually deteriorates, making the material more prone to decomposition at high temperatures. Consequently, both materials exhibit a decline in their overall heat resistance over time.

## 3. Simulation of Decomposition Reactions Based on Molecular Dynamics

### 3.1. Construction of Silicone Rubber Molecular Model

To systematically investigate the thermal decomposition behavior of high-temperature vulcanized (HTV) and liquid silicone rubber (LSR) during aging, molecular models were constructed and analyzed using Materials Studio. This platform integrates multiple molecular simulation methods, enabling accurate representation of microscopic structural features and efficient quantitative evaluation of polymer properties. First, within the Visualizer module, a single-chain molecular model of methyl vinyl silicone rubber was manually constructed based on its molecular formula. For the HTV silicone rubber model, the degree of polymerization of methyl siloxane was set to 20, while that of vinyl groups was 1 [17]. For the LSR model, the corresponding degrees of polymerization were 10 and 2, respectively [18]. Subsequently, the Amorphous Cell module was employed to construct two silicon–oxygen chains and to simulate cross-linking within an amorphous cell. During this process, the vinyl groups on the side chains exhibited the highest reactivity and acted as cross-linking sites in the vulcanization reaction, forming C–C bonds between polymer chains. After completion of the cross-linking process, the configuration with the lowest energy was selected as the optimized initial structure for subsequent simulations.

First, the Amorphous Cell module in Materials Studio is employed to assign the force field and charge distribution, followed by geometric optimization of the initially constructed HTV and LSR models to minimize their potential energy. Subsequently, cyclic annealing is performed under the NVT ensemble for 2000 ps, with the temperature ranging from 300 K to 600 K. The lowest-energy configuration obtained during the annealing process is selected as the optimized structure. Finally, molecular dynamics simulations are carried out on the annealed models using the NPT ensemble at 298 K with a total of 10,000 simulation steps. Upon completion of the simulations, the calculated density of the HTV silicone rubber system is 1.021 g/cm^3^, which falls within the experimentally reported range of 0.98–1.20 g/cm^3^ [19], indicating that the constructed HTV model is consistent with realistic conditions. Similarly, the density of the LSR silicone rubber system is determined to be 1.011 g/cm^3^, which agrees well with the reference range of 0.99–1.03 g/cm^3^ for force field-based simulations [20]. This result confirms the reliability and rationality of the LSR silicone rubber model established in this study. The molecular models of HTV and LSR silicone rubber developed in this study are idealized, simplified representations of polymer matrix systems. These models focus solely on the main chain structure of the silicone rubber and the differentiated cross-linking methods, excluding the complex components present in actual engineering materials, such as inorganic fillers, processing aids, additives, residual catalysts, and trace impurities. As a result, these models cannot fully replicate the multi-component system found in real-world service casings. The simulation results are primarily intended to qualitatively elucidate the fundamental mechanisms of thermal decomposition and structural degradation of silicone rubber at the molecular level, providing insight into the experimental phenomena from a microscopic perspective. These results should not be interpreted as a direct basis for a fully quantitative comparison with the actual aged materials.

### 3.2. Reaction Dynamics Simulation Based on the ReaxFF Reaction Force Field

This study employs the ReaxFF module implemented in LAMMPS [21] to perform reactive molecular dynamics simulations of the thermal decomposition of silicone rubber. ReaxFF, a reactive force field designed for molecular dynamics simulations, enables the dynamic formation and breaking of chemical bonds, thereby facilitating the investigation of microscopic structural evolution in complex reactive systems [22]. High-temperature ReaxFF simulations are adopted as an acceleration strategy to efficiently capture bond dissociation sequences and reaction pathways within a computationally feasible time scale. It should be noted that these simulations are not intended to directly reproduce the long-term aging process of silicone rubber, nor to match real aging time scales. Instead, the focus is placed on elucidating the staged molecular evolution and identifying the intrinsic degradation mechanisms that are independent of time scale. The high-temperature vulcanized (HTV) and liquid silicone rubber (LSR) models constructed in this study are converted into data files suitable for reactive dynamics calculations. Simulations are conducted under the NVT ensemble with real units, charge-based atomic types, periodic boundary conditions, and the CHONSSi.ff reactive force field. The temperature is varied from 300 K to 5000 K. During the decomposition process, simulations are performed over time scales ranging from picoseconds to nanoseconds. The stabilization point of the reaction is determined based on the evolution of product species. The total simulation time is set to 200 ps with a time step of 0.1 fs, and thermodynamic data as well as reaction states are recorded every 100 steps. It is important to emphasize that the selected simulation temperature, system size, and boundary conditions do not affect the fundamental degradation pathways of silicone rubber. In this study, all comparative analyses were performed under a unified simulation temperature, model scale, and periodic boundary conditions. Owing to the limited system size inherent in molecular simulations, the use of elevated-temperature acceleration, and the implementation of periodic boundary conditions, these simulation settings may influence the statistical characteristics of reactions, product distributions, and reaction pathway preferences. Nevertheless, within the standardized modeling and computational framework, the simulations are capable of effectively capturing representative microscopic reaction processes and structural evolution behaviors during the thermal degradation of silicone rubber, thereby satisfying the requirements for comparative analysis of degradation mechanisms across different systems.

### 3.3. Analysis of Decomposition Pathways

**Figure 6:**
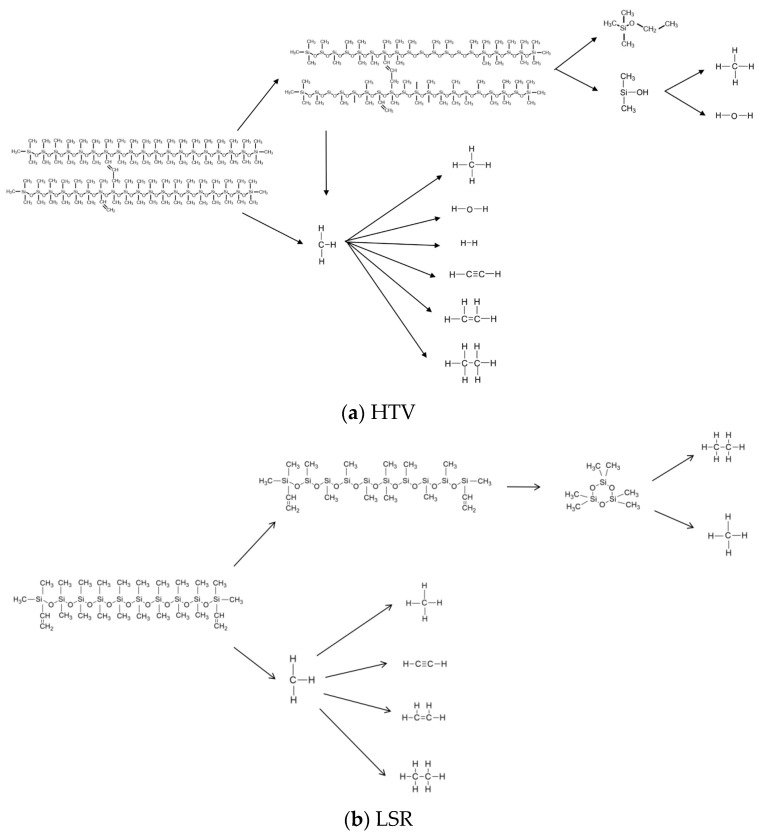
Silicone rubber molecular reaction path diagram.

This study systematically investigates the electrothermal cracking pathways and formation mechanisms of decomposition products by analyzing the dynamic behavior of two types of silicone rubber molecules during thermal decomposition. The thermal cracking pathway of HTV silicone rubber is illustrated in Figure 6a. In the decomposition pathway of HTV silicone rubber with the molecular formula C_90_H_264_O_40_Si_42_, the Si–C bonds along the polymer backbone are initially cleaved, accompanied by the detachment of methyl groups. This process generates high-order siloxane fragments with the composition C_55_H_117_O_23_Si_40_ and methyl radicals CH_3_. The methyl radicals subsequently undergo rearrangement and secondary reactions, forming small-molecule products such as H_2_O, H_2_, C_2_H_2_, C_2_H_4_, and C_2_H_6_. At this stage, further demethylation occurs within the high-order siloxane fragments with the composition C_55_H_117_O_23_Si_40_. As the reaction progresses, additional cleavage of the siloxane backbone leads to the formation of smaller siloxane species and hydroxyl-containing siloxane intermediates. These hydroxyl-containing intermediates are thermally unstable and undergo subsequent demethylation and dehydroxylation under high-temperature conditions, ultimately producing small-molecule species such as CH_4_ and H_2_O. The thermal cracking pathway of LSR silicone rubber is shown in Figure 6b. In the LSR decomposition of C_22_H_58_O_9_Si_10_, Si–C bonds along the main chain break first, releasing methyl groups and forming cyclic oligomers [(CH_3_)_2_SiO]_3_. The liberated methyl radicals then participate in further reactions, producing small molecules such as CH_4_, C_2_H_2_, C_2_H_4_, and C_2_H_6_. Subsequently, the cyclic oligomers themselves undergo ring-opening, generating additional small-molecule products including CH_4_ and C_2_H_6_. The thermal decomposition reaction pathways presented in this study are representative examples obtained from the simulation process and are used to illustrate key microscopic behaviors, including molecular chain scission, group dissociation, and the formation and evolution of cyclic products. Owing to the stochastic nature of reaction kinetics, the thermal decomposition of silicone rubber proceeds through multiple competing reaction channels. The pathways described herein, such as bond cleavage, radical rearrangement, and ring-opening of cyclic oligomers, are not exhaustive but represent the dominant reaction routes that occur with higher frequency in the simulation system. Furthermore, in conjunction with existing literature on the thermal degradation of silicone rubber, the observed microscopic behaviors—such as the preferential cleavage of Si–C bonds, the formation of side-group radicals, and the ring-opening decomposition of cyclic structures—are consistent with previously reported mechanisms, thereby supporting the rationality of the reaction pathways observed in the simulation.

### 3.4. Analysis of Decomposition Products

**Figure 7:**
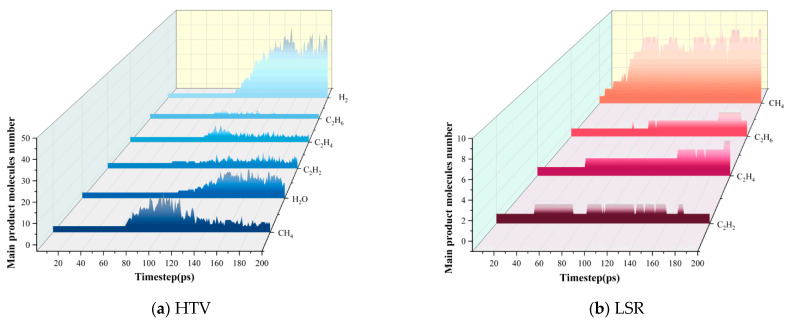
Graph showing the quantity of silicon rubber decomposition products.

Figure 7 illustrates the temporal evolution of the main pyrolysis products of silicone rubber. The release sequence and concentration evolution of products differ significantly between the two materials, which is fundamentally governed by differences in matrix network density, chain segment binding strength, and competitive reaction pathways. For the HTV system, the molecular network remains relatively intact at the initial stage of pyrolysis, with limited bond scission and side-group dissociation, resulting in a low yield of small-molecule products. As the reaction proceeds, molecular chain constraints gradually weaken, and backbone cleavage, side-group detachment, and radical reactions become increasingly pronounced, leading to substantial formation of CH_4_, H_2_, light hydrocarbons, and oxygen-containing species. In particular, methyl side groups are more prone to dissociation and rearrangement, resulting in a more pronounced increase in CH_4_ and H_2_. In the later stage, the depletion of reactive sites slows down further degradation, and ring-opening reactions of cyclic oligomers gradually diminish. Consequently, the concentration of most light hydrocarbons decreases, while H_2_O production—affected by oxidation and structural rearrangement—exhibits a reduced growth rate and a dynamic equilibrium behavior. In comparison, the dense cross-linked network and stronger intermolecular interactions in LSR significantly alter the pyrolysis reaction pathways. At the initial stage, overall reaction activity is suppressed; however, local methyl side-group dissociation occurs preferentially, leading to the early formation of CH_4_. During the intermediate stage, chain segment activation and localized bond scission intensify, resulting in the concurrent accumulation of various light hydrocarbons. Owing to the higher structural stability of the Si–O backbone, decomposition of the LSR matrix proceeds more gradually, with lower small-molecule yields compared to HTV. In the later stage, as reactive groups become depleted and ring dissociation approaches saturation, most small-molecule species decline. CH_4_, governed by the slow cleavage of stable side groups, exhibits a slower formation rate and maintains a relatively stable concentration.

## 4. Comparative Analysis of Physical and Chemical Properties of Silicone Rubber Aging

### 4.1. Glass Transition Temperature

The glass transition temperature (Tg) is the temperature at which a polymer undergoes a transition from a glassy to a highly elastic state. It is commonly used to characterize the stability of polymer-based macromolecules [23]. As the temperature exceeds Tg, the conversion between these two states results in a significant decrease in thermodynamic properties, along with notable changes in volume and density. In general, for a given pure polymer matrix, a higher Tg indicates a stronger restriction on the movement of molecular chain segments, thereby slowing the development of processes such as chain segment slip and thermal oxygen-induced degradation.

**Figure 8:**
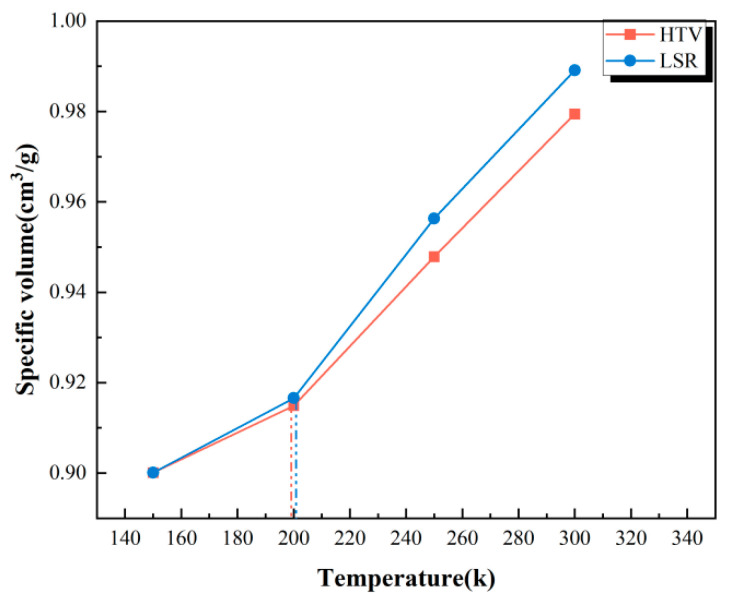
Glass transition temperature.

In this study, the Tg was calculated using the volume–temperature curve method [24]. Specifically, the average density values of the final 100 ps of the NPT ensemble simulation were extracted at each temperature, and the reciprocal of these values was taken to obtain the specific volume. Linear fitting was applied to the data points of each temperature segment to determine the intersection point, which represents the Tg. Figure 8 presents the results for the glass transition temperature of silicone rubber. Under the idealized matrix model conditions, the Tg of HTV and LSR was found to be approximately 200 °C, with LSR exhibiting a slightly higher Tg than HTV. This result qualitatively reflects the characteristics of molecular chain movement, indicating that the molecular chain segments in LSR are more strongly restrained, leading to a more gradual process of structural relaxation and degradation under the simulation conditions. However, the macroscopic thermal resistance of actual engineering materials is influenced by multiple factors, including filler systems, formulation components, and crosslinking processes. 

### 4.2. Molecular Chain Movement Characteristics

To investigate the thermal decomposition behavior of HTV and LSR under different aging conditions, structural models at different stages of thermal decomposition (5 ps and 60 ps) were selected to represent different degrees of decomposition at the atomic scale. These simulation results were qualitatively compared with the experimentally observed microstructural evolution of field-aged samples to analyze the influence of structural changes on thermal stability. The mean square displacement (MSD) is a key metric for quantitatively describing the movement characteristics of the molecular chain’s center of mass. It directly reflects both the diffusion ability and the extent of motion restriction of the molecular chain segments [25]. In general, within the same pure polymer system, a lower MSD value indicates a higher degree of restriction on molecular chain movement, leading to a slower overall relaxation rate of the network structure. Therefore, the MSD can serve as a microscopic indicator to assess the evolution of the matrix structure. To ensure statistical accuracy and eliminate non-equilibrium effects during the simulation relaxation stage, the trajectory data from the final 30 ps of the simulation, once the system has fully relaxed and reached thermodynamic stability, are selected for MSD calculation and curve fitting.

**Figure 9:**
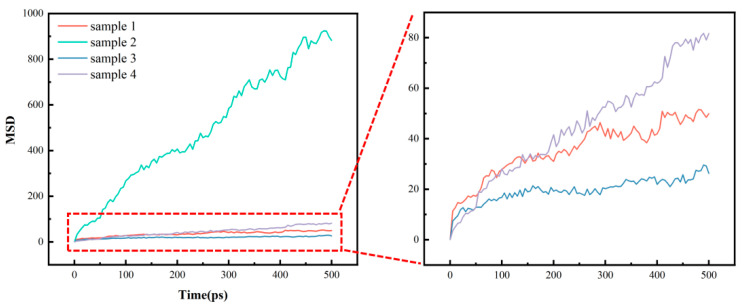
The MSD curve at a temperature of 300K.

Figure 9 presents the MSD curves for the HTV and LSR models at 300 K. A comparison of the four MSD curves reveals that the MSD value of sample 3 is consistently lower than that of sample 1, and the MSD value of sample 4 is consistently lower than that of sample 3. In contrast, the MSD value of sample 2 is significantly higher than that of sample 1, and the MSD value of sample 4 is higher than that of sample 3. These results suggest that within the idealized pure matrix simulation system, LSR molecular chain segments experience stronger constraints, resulting in weaker diffusion of the chain segments. The aging effect further diminishes the molecular network’s constraint ability, thereby increasing the activity of the chain segments and reducing the regularity of the silicone rubber matrix structure. It is important to note that this conclusion pertains solely to the simplified molecular model, which excludes fillers and additives, and serves to reveal the inherent chain segment evolution behavior of silicone rubber. The macroscopic aging and stability of actual engineering materials are influenced by various factors, including filler composition, cross-linking processes, impurities, and multi-field environmental interactions. 

### 4.3. Cohesion Energy Density

Cohesion energy density (CED) refers to the energy required to overcome the intermolecular forces within a unit volume and is used to quantify the strength of these interactions. In an idealized pure polymer matrix system, a higher CED typically indicates stronger intermolecular forces, more closely packed molecular chains, and a greater restraining effect on the movement of chain segments. CED can therefore serve as a microscopic reference for evaluating the aggregation state and inherent stability of the matrix structure. In this study, the CED of the silicone rubber system within a composite framework was calculated at 300 K using the Forcite module. All calculations were performed using the COMPASS III force field, which is consistent with the cracking simulation to ensure a fair comparison.

**Figure 10:**
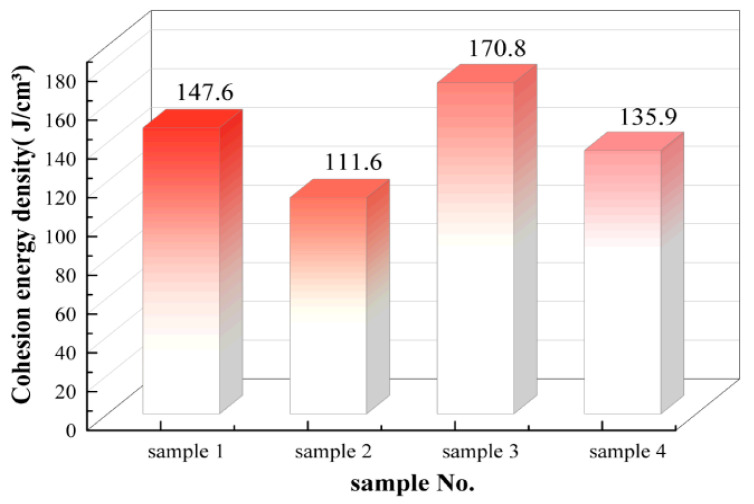
Cohesion energy density.

Figure 10 presents the CED values for the HTV and LSR models at 300 K. The CED of samples 3 and 4 is significantly higher than that of samples 1 and 2. Additionally, the CED of sample 2 is slightly lower than that of sample 1, and the CED of sample 4 is slightly lower than that of sample 3. These results suggest that the LSR model exhibits overall stronger intermolecular interactions and a more densely packed microscopic structure compared to the HTV model. Furthermore, within the same material system, the CED values of the aged samples decrease to some extent, indicating that aging progressively weakens intermolecular interactions, leading to a looser aggregation structure of the matrix.

### 4.4. Free Volume Fraction

**Figure 11:**
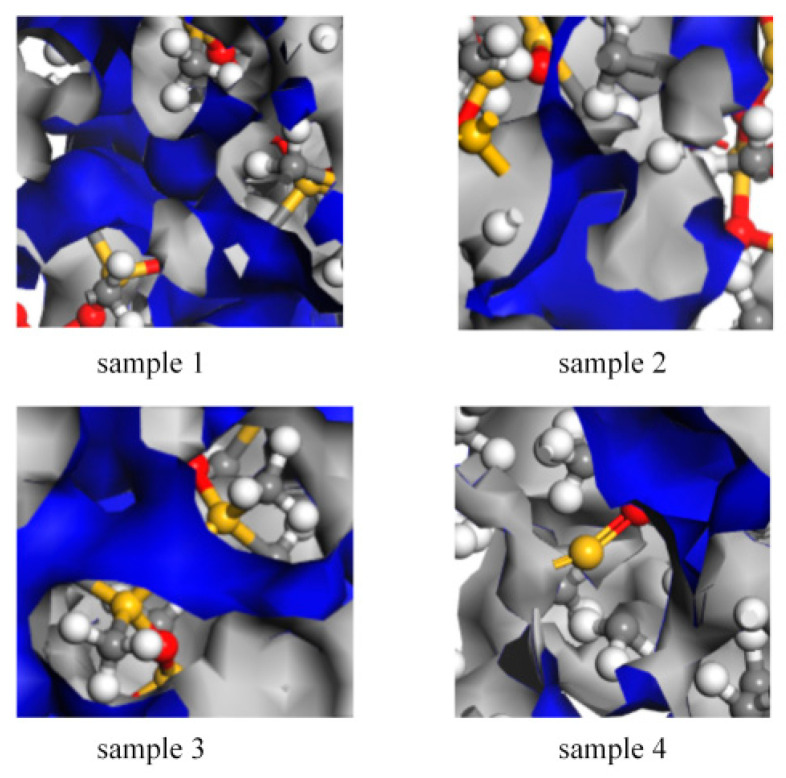
Model free volume distribution.

The free volume fraction (FFV) indicates the degree of molecular chain packing and the size of the available free space within the material [26]. In a pure matrix simulation system, a lower FFV value suggests more compact molecular packing, with greater restriction on the free space available for chain segment movement. This can be considered a reflection of the matrix’s inherent resistance to structural degradation at the microscopic level. In this study, the free volume distribution was calculated with high precision using a 1.0 Å probe in Materials Studio. The resulting model of the free volume distribution is presented in Figure 11.

**Figure 12:**
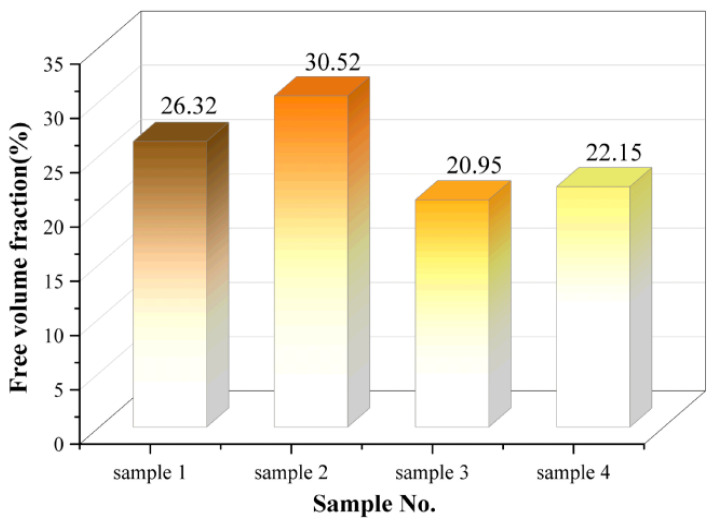
Free volume fraction.

Figure 12 presents the free volume fraction test results for the HTV and LSR models. The FFV of samples 3 and 4 is significantly lower than that of samples 1 and 2. Additionally, the FFV of sample 2 is higher than that of sample 1, and the FFV of sample 4 is higher than that of sample 3. Overall, the FFV of the LSR model is lower than that of the HTV model. Under the simplified simulation conditions of this study, these results suggest that the LSR matrix is more densely packed, with more limited molecular chain mobility. In contrast, for the same material system, the FFV of the aged models has increased to varying extents, indicating that aging disrupts the dense network structure of silicone rubber, increasing the available space for chain segment movement. This promotes the relaxation of molecular chains and contributes to structural deterioration.

## 5. Analysis of the Mechanism Linking Structural Changes and Thermal Decomposition Properties After the Aging of Silicone Rubber

To thoroughly investigate the thermal decomposition characteristics of HTV and LSR under different aging conditions, this study selects two types of silicone rubber with structural models at different thermal decomposition stages (5 ps and 60 ps). A qualitative comparison is made of the microscopic structural states of the samples under various aging degrees, aimed at analyzing the impact of microscopic structural evolution on thermal stability. The ReaxFF simulation employs high-temperature accelerated thermal decomposition on a picosecond timescale, which does not directly correlate with the long-term aging observed in field conditions. Therefore, this paper uses the structural evolution at different thermal decomposition stages to qualitatively compare the microscopic deterioration trends resulting from actual aging, providing mechanistic insights to complement macroscopic experimental results. By combining scanning electron microscopy images of aged silicone rubber samples with thermogravimetric analysis data, it is evident that LSR and HTV exhibit distinct differences in thermal degradation behavior and surface deterioration characteristics. A comprehensive analysis of multiple thermogravimetric indicators and microscopic morphological changes suggests that LSR generally demonstrates better thermal degradation resistance. However, as aging progresses, both types of silicone rubber exhibit a certain degree of attenuation in their thermal stability.

To investigate the microscopic evolution of thermal decomposition behavior, molecular models were constructed and analyzed using the Materials Studio platform. Reaction molecular dynamics simulations based on the ReaxFF force field, as implemented in LAMMPS, were employed to study the decomposition behavior of silicone rubber. Under idealized pure matrix conditions, LSR exhibits a higher glass transition temperature, suggesting stronger constraints on molecular chain segments, which may effectively suppress segmental activation and degradation. Under the same aging gradient, the mean square displacement (MSD) of the LSR model consistently remains lower, indicating reduced chain segment diffusion and molecular mobility. Aging enhances chain segment mobility in both types of silicone rubber, leading to a relaxation of the microscopic network structure. Meanwhile, the LSR model shows a higher cohesive energy density, which may reflect stronger intermolecular interactions and a more compact packing structure. However, continuous aging gradually weakens these intermolecular forces and reduces the overall binding strength of the system. Furthermore, LSR exhibits a lower free volume fraction and higher packing density, which may help retard molecular chain relaxation and structural degradation. As aging progresses, the initially dense network is disrupted, free volume increases, and the space available for chain segment motion expands, potentially accelerating the deterioration of the matrix structure.

The molecular dynamics simulations presented above are based on a simplified polymer model without fillers. From the perspectives of molecular chain motion, intermolecular interactions, and aggregated structure, they qualitatively indicate the intrinsic structural differences between the two silicone rubber matrices, as well as the effects of aging on their microscopic evolution behavior. The simulated microscopic trends are generally consistent with the macroscopic degradation behaviors observed in SEM morphology and thermogravimetric analysis, providing possible mechanistic insights that are consistent with the experimental results. Compared with the HTV system, LSR exhibits stronger intrinsic chain constraints, more pronounced intermolecular interactions, and a more compact packing structure, which may contribute to its superior resistance to thermal degradation. Aging progressively disrupts the cross-linked network and weakens intermolecular interactions in silicone rubber, potentially leading to a reduction in chain segment constraints and structural density, and ultimately to a gradual decline in thermal stability and degradation resistance. However, it should be noted that these simulations do not account for complex components such as fillers, additives, or residual impurities present in practical engineering materials; therefore, the conclusions are limited to idealized polymer matrices. By integrating microscopic dynamic parameters with macroscopic experimental characterization, the underlying mechanisms of aging-induced degradation in silicone rubber can be further explored, providing a theoretical basis for future modification and optimization aimed at improving aging resistance.

## 6. Conclusions

This study combined macroscopic characterization and ReaxFF molecular dynamics simulations to investigate the thermal decomposition behaviors and possible microscopic mechanisms of HTV and LSR silicone rubbers under varying aging conditions. Key thermodynamic parameters were also calculated to explore the relationship between molecular structure and material performance. The main conclusions are summarized as follows:

(1) Thermogravimetric analysis indicates that LSR exhibits superior thermal stability compared to HTV, which is supported by higher 5 percent weight loss temperatures, elevated maximum decomposition rate temperatures, and greater high-temperature residual mass. The thermal stability of both materials gradually decreases with increasing aging degree.

(2) ReaxFF simulations indicate that HTV and LSR silicone rubbers undergo distinct thermal decomposition stages and exhibit different product evolution behaviors. HTV exhibits a steady increase in the formation of small-molecule products, whereas LSR shows slower and more stable product generation.

(3) Under the simplified matrix model, LSR exhibits a higher glass transition temperature, stronger intermolecular interactions, and a more compact molecular structure, which may retard degradation. Aging enhances molecular mobility and increases free volume, potentially accelerating structural deterioration in both materials.

Although the high-temperature accelerated molecular dynamics simulations based on the ReaxFF force field provide potentially useful insights into the structural evolution and thermal decomposition characteristics of silicone rubber, there are several significant limitations to this approach. First, the timescale is significantly compressed due to the high-temperature acceleration, which introduces discrepancies when compared to real-world aging processes. As a result, the simulated reaction pathways and product distributions may not fully reflect the long-term degradation mechanisms observed in actual service conditions. Additionally, the chemical model used in this study simplifies the polymer matrix by excluding the presence of fillers, additives, and other components typically found in engineering materials. This simplification may affect the accuracy of the simulation results and limit their direct applicability to practical materials. The choice of the ReaxFF force field itself introduces another layer of complexity, as the accuracy of the results is dependent on the quality of the force field parameters and their fitting to the specific system under study. Furthermore, the simulations are conducted in an idealized, isolated environment without introducing the oxidative atmosphere that is typically present in real-world aging conditions. The absence of oxidation interactions may lead to an incomplete representation of the material’s behavior under natural aging processes. Consequently, the simulations cannot directly map to the aging mechanisms in actual service environments, nor can they establish a one-to-one correspondence with the performance of real-world materials. These limitations imply that while the simulation results provide potentially useful mechanistic insights into the decomposition behavior of silicone rubber, they cannot be considered definitive for determining the true aging mechanisms under practical conditions. The reaction pathways and product distributions observed in the simulations should be regarded as qualitative approximations that require further validation through experimental studies.

## Figures and Tables

**Table 1:** Table of Specific Aging Environment Settings for Silicone Rubber Samples.

Sample No.	Type	Sampling Location	Degree of Aging
1	HTV	Device end umbrella skirt	mild
2	HTV	Device end main body	Severe
3	LSR	High-voltage end insulation sleeve body	mild
4	LSR	High-voltage end umbrella skirt	Severe

## Data Availability

The original contributions presented in this study are included in the article. Further inquiries can be directed to the corresponding author.
